# Conserved microstructure of the *Brassica* B Genome of *Brassica nigra* in relation to homologous regions of *Arabidopsis thaliana*, *B. rapa* and *B. oleracea*

**DOI:** 10.1186/1471-2164-14-250

**Published:** 2013-04-15

**Authors:** Zahra-Katy Navabi, Terry Huebert, Andrew G Sharpe, Carmel M O’Neill, Ian Bancroft, Isobel AP Parkin

**Affiliations:** 1 Agriculture and Agri-Food Canada, 107 Science Place, Saskatoon, SK S7N 0X2, Canada; 2 DNA Technologies Laboratory, 110 Gymnasium Place, Saskatoon, SK S7N 0W9, Canada; 3 John Innes Centre, Norwich Research Park, Colney, Norwich NR4 7UH, UK

**Keywords:** Brassiceae, *Brassica nigra*, Sequence analyses, Speciation, Genome organization, Collinearity, Divergence time, Inversion

## Abstract

**Background:**

The *Brassica* B genome is known to carry several important traits, yet there has been limited analyses of its underlying genome structure, especially in comparison to the closely related A and C genomes. A bacterial artificial chromosome (BAC) library of *Brassica nigra* was developed and screened with 17 genes from a 222 kb region of *A. thaliana* that had been well characterised in both the *Brassica* A and C genomes.

**Results:**

Fingerprinting of 483 apparently non-redundant clones defined physical contigs for the corresponding regions in *B. nigra*. The target region is duplicated in *A. thaliana* and six homologous contigs were found in *B. nigra* resulting from the whole genome triplication event shared by the Brassiceae tribe. BACs representative of each region were sequenced to elucidate the level of microscale rearrangements across the *Brassica* species divide.

**Conclusions:**

Although the B genome species separated from the A/C lineage some 6 Mya, comparisons between the three paleopolyploid *Brassica* genomes revealed extensive conservation of gene content and sequence identity. The level of fractionation or gene loss varied across genomes and genomic regions; however, the greatest loss of genes was observed to be common to all three genomes. One large-scale chromosomal rearrangement differentiated the B genome suggesting such events could contribute to the lack of recombination observed between B genome species and those of the closely related A/C lineage.

## Background

Black mustard (*Brassica nigra* (L.) Koch, 2*n* = 16, BB genome) represents the diploid *Brassica* B genome. It is an outcrossing oilseed species [1] which can also be used as a condiment [2]. However, it is a relatively minor crop compared with the closely related mustard species *Brassica juncea* (L.) Czern (AABB genome) and *Brassica carinata* Braun (BBCC genome), which are allotetraploid species that share the B genome [1]. Most studies within the Brassiceae have focused on the widely cultivated *Brassica* species, *Brassica rapa* L. (AA genome), *Brassica oleracea* L. (CC genome), and *Brassica napus* L. (AACC genome) [3]. However, the diploid *Brassica* B genome is considered to be an important source of useful genes in *Brassica* breeding, including drought tolerance, disease resistance, and oil seed quality [4-9]. In order to exploit the variation found within the B genome for the breeding of other *Brassica* oilseeds, an understanding of the relationship between the three *Brassica* genomes is required.

Although there has been extensive evidence of pairing and recombination between the A and C *Brassica* genomes [10-12] the B genome appears unusual since no or limited recombination between the B genome and its related A and C homologues has been observed [13-17]. The reason for this difference can be hypothesized as either the result of genetic factors regulating homologous pairing in the B genome similar to those suggested for the A and C genomes [18-21] or significant structural divergence of the B genome relative to the A and C genomes. Although it has been suggested through limited sequence and comparative mapping data that the B genome may have diverged to such an extent that the lack of recombination can be explained [22], questions still arise as to accuracy of this assertion. Uncovering the level of homology at the micro level between the three *Brassica* diploid genomes should provide insights into the true relationship between the genomes.

The cultivated *Brassica* species are closely related to the fully sequenced model plant *Arabidopsis thaliana*, [23] since the *Brassica* and Arabidopsis lineages diverged between 14.5 and 20 million years ago (Mya) [24]. It is believed that *B. nigra* evolved from the same polyploidy events, and resultant paleohexaploid, which took place around 7.9 to 14.6 Mya within the *Brassica* lineage. Although there are good estimates placing the separation of the *B. rapa* (A genome) and *B. oleracea* (C genome) at 3.7 Mya [25], there is little data to age the separation of the B genome from the A/C lineage [26]. Comparative mapping has identified blocks of conserved gene content and order between the *A. thaliana* genome and the *Brassica* A and C genomes of *B. napus*; where 21 ancestral segments of the *A. thaliana* genome could be replicated and rearranged to represent the *Brassica* genomes [27]. This work and further mapping in related species was used to propose an ancestral karyotype, reconstructed from 24 conserved blocks, A-X, for the entire Brassicaceae family [28]. Physical mapping of the regions homologous to a 222 kb region of *A. thaliana* chromosome 4 in *B. oleracea*[29], *B. rapa*[30] and *B. napus*[31] showed highly conserved collinearity of the orthologous regions in the three genomes and corroborated the triplicated nature of the diploid *Brassica* genomes. Previous sequence-level studies in *B. oleracea*[32], *B. rapa*[33] and *B. napus*[34] have clarified aspects of genome evolution and organization in *Brassica* by exploiting the close relationship with the genome of the model plant *A. thaliana*.

Physical mapping can provide an accurate representation of a genome when sequence is not available. A number of large insert libraries, the foundation for most physical maps, have been developed for the *Brassica* A and C genome species and have been exploited to facilitate map-based gene cloning for traits of interest and to provide insights into the evolutionary mechanisms that have formed these complex genomes [29-32,35-39]. In order to provide insights into the relatively uncharted *Brassica* B genome we have developed a large insert library for *B. nigra* and have used this tool to describe the physical organisation of six regions of the B genome of *B. nigra* homologous to the same 222 kb region of *A. thaliana*, which was exploited previously to uncover aspects of genome organisation and evolution in the *Brassica* A/C genomes [29,30,32,34]. We compared these orthologous regions of the B genome with the equivalent regions of *A. thaliana*, and the *Brassica* A and C genomes at the sequence level and report highly conserved gene content and order across the three genomes. An earlier divergence time separated the B genome from the A and C Brassica lineage by some 3 million years, which is barely reflected in the level of observed genome fractionation.

## Results

### BGH BAC library characteristics

In order to facilitate comparative mapping among the *Brassica* species a BAC library was constructed from a doubled haploid (DH) *B. nigra* line (No100). The *B. nigra* library (BGH) was generated from *Hind*III digested genomic DNA and, includes 85,248 clones arrayed in 384 well plates. One clone was randomly selected from each of 100 plates. Restriction digestion of BAC DNA and resolution with pulse field gel electrophoresis allowed the average insert size to be estimated at 130 kb. There were no empty clones identified among the 100 selected. In order to accurately assess the number of empty clones, 20 plates (7,680 clones) were gridded in replicate onto medium containing IPTG and X-gal, which resolved 141 (1.8%) empty clones. The library should provide an approximate 17.5X coverage of the B genome, which is estimated to be 632 Mb [40].

### Identifying the B genome BACs homologous to the *A. thaliana* target region

**Figure 1:**
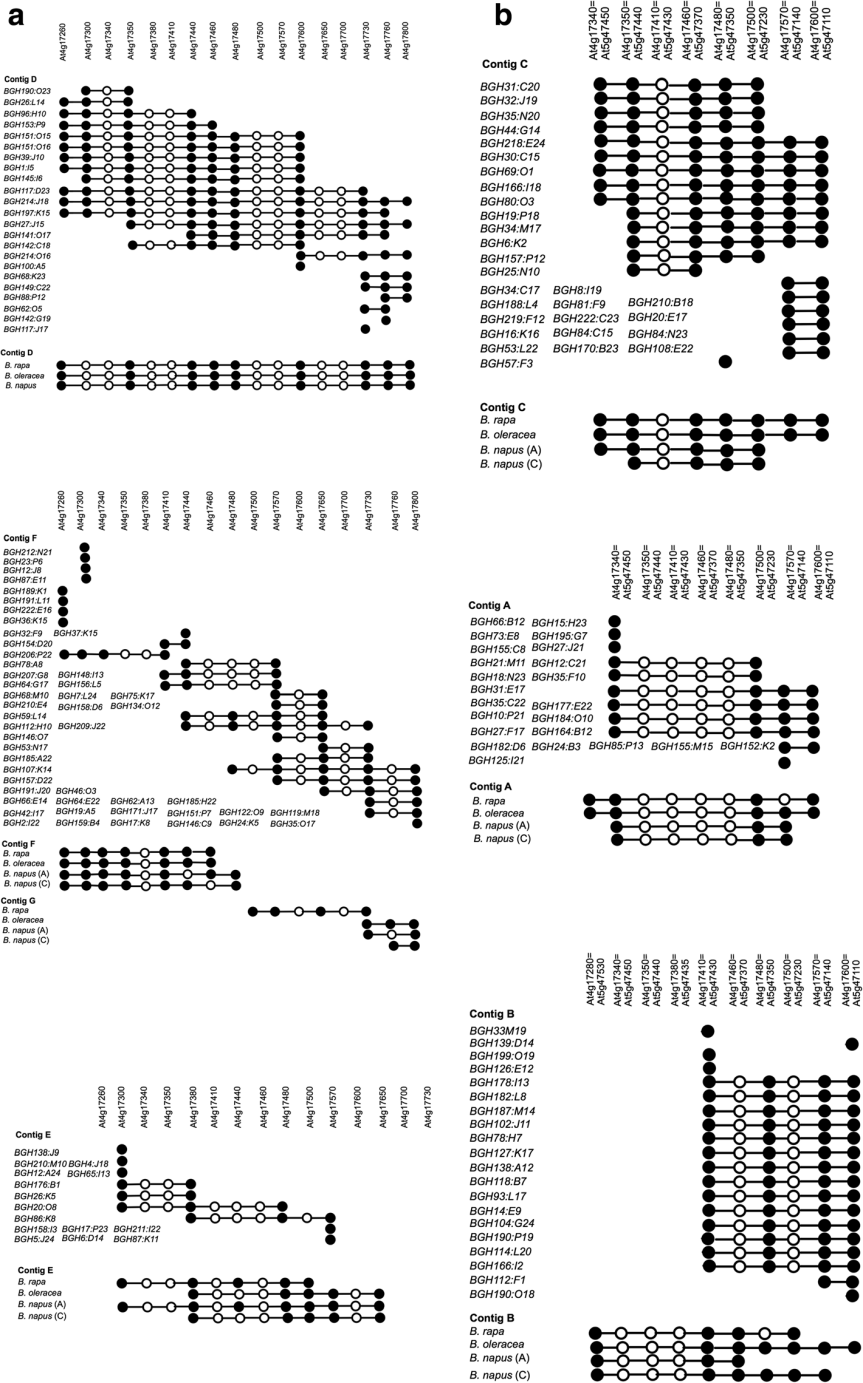
***Brassica nigra *contigs homologous to the target region.** Contigs were assembled based on common *Hind*III digestion patterns identified by two or more genes from the target region. Closed circles show hybridisation of the gene specific probe and open circles indicate no hybridisation. Contigs aligned to *A. thaliana* chromosome 4 (**a**) and chromosome 5 (**b**).

We targeted the 222 kb region on *A. thaliana* chromosome 4, which had been studied previously in *B. rapa*[30], *B. oleracea*[29] and *B. napus*[31] and is also partially duplicated on *A. thaliana* chromosome 5, resulting from the known α-duplication event. Seventeen probes representing *A. thaliana* genes from this region were hybridised to high-density colony filters of the BGH library. The probes identified 18–207 BACs each, with 207 BAC clones being recognised by two or more probes. The resulting 1,110 BAC clones were end-sequenced, with successful sequence being acquired from 851 clones. These sequence data were compared against each other and against the *A. thaliana* gene complement using BLASTN, which identified 483 potentially non-redundant BAC clones for further analyses. The resultant banding patterns of the *Hind*III digested BAC clones hybridised with the gene specific probes were studied to confirm the initial data and to find those clones with shared loci (Additional file 1: Table S1 and Figure 1). Of the 483 selected BACs a total of 362 clones showed strong positive hybridisation for the 17 gene-specific probes while 121 from this subset did not. The number of BACs and loci identified for each gene specific probe is reported in Table 1, with the number of homologous loci ranging between 1 and 4 for each *A. thaliana* gene.

**Table 1:** **Details of probes used to screen *B. nigra *BAC library and the results from the hybridisation data**

**Gene specific probe**	**Old code**	**Number of positive clones**	**Number of loci**	**Mean redundancy**
At4g17260	DL 4665	28	1	28.0
At4g17300	DL 4685	37	1	37.0
At4g17340	DL 4705	77	1	77.0
At4g17350	DL 4710	54	2	27.0
At4g17380	DL 4725	17	2	8.5
At4g17410	DL 4740	43	2	21.5
At4g17440	DL 4755	83	1	83.0
At4g17460	DL 4765	51	3	17.0
At4g17480	DL 4775	35	4	8.7
At4g17500	DL 4785	40	1	40.0
At4g17570	DL 4820	58	2	29.0
At4g17600	DL 4835	107	2	53.5
At4g17650	DL 4860	21	1	21.0
At4g17700	DL 4885	10	3	3.3
At4g17730	DL 4900	67	1	67
At4g17760	DL 4915	19	2	9.5
At4g17800	DL 4935	85	3	28.3

Contigs of overlapping BAC clones were assembled based on common digestion patterns observed for two or more genes. In addition, all 483 BAC clones were subjected to SNaPshot high information content fingerprinting (HICF) and the FPC software was used to assemble contigs [38,41,42]. In FPC it is recommended to start building contigs at high stringency to prevent chimeric joining of duplicated regions and to iteratively lower the stringency to avoid gaps in the resultant physical map [43]. Therefore we started with a cut off value of 1 × 10^-35^ for automatic contig assembly and used the “DQer” function to break up Q contigs (contigs containing more than 10% Questionable clones) which resulted in 16 contigs and 62% (302) singletons. The stringency was lowered in a stepwise manner and the project finished with an optimal lower cut off value of 1 × 10^-15^, which resulted in 35 contigs with 38% (186) singletons that could not be incorporated into other contigs (Additional file 1: Table S1). This resulted in an assembly with a low number of Q clones (0–5 clones in each contig). Six B genome contigs which had the highest number of overlapping BACs (Figure 1) and were in agreement with the Southern hybridisation results were considered representative of the *A. thaliana* target region. The assembled contigs appeared to follow the order of the genes as represented in the *A. thaliana* genome (Additional file 1: Table S2 and Figure 1). Three B genome regions were homologous to *A. thaliana* chromosome 5 (contigs A, B and C) while the other three were homologous to *A. thaliana* chromosome 4 (contigs, D, E and F), and the contigs were named accord to the previous analysis of the Brassica A/C genomes [29,30]. The contig assignments were based on the presence or absence of eight genes (At4g17260, At4g17300, At4g17380, At4g17440, At4g17650, At4g17750, At4g17760, and At4g17800) that are present in the chromosome 4 region, but not in the chromosome 5 region [29,44]. At this macro-level the only apparent major difference between the *Brassica* B genome and A and C genomes was the joining of contigs F and G in the B genome, which are physically separated in the A/C genomes (Figure 1).

### Comparative organisation at the sequence level

BACs were selected for sequencing in order to represent each of the six contiguous regions identified as being homologous to the *A. thaliana* region (Figure 1). The list of BAC clones sequenced, and their characteristics are detailed in Table 2.

**Table 2:** **List of Sequenced *B. nigra *BAC clones**

***B. nigra *BAC**	**Genome segment**	**Size bp**	**Accession number**
BGH184:O10	*B. nigra* Contig A	116,795	KC96003
BGH31:E17	*B. nigra* Contig A	257,762	KC95996
BGH93:L17	*B. nigra* Contig B	258,014	KC96000
BGH8:I19	*B. nigra* Contig C	127,829	KC95992
BGH34:M17	*B. nigra* Contig C	158,918	KC95997
BGH24:O18	*B. nigra* Contig D	139,013	KC95995
BGH214:J17	*B. nigra* Contig D	384,919	KC96005
BGH214:O16	*B. nigra* Contig D	140,361	KC96006
BGH20:O8	*B. nigra* Contig E	142,538	KC95994
BGH12:A24	*B. nigra* Contig E	121495	KC95993
BGH148:I13	*B. nigra* Contig F	203,952	KC96002
BGH59:L14	*B. nigra* Contig F	169,401	KC95998
BGH64:E22	*B. nigra* Contig F	124,814	KC95999
BGH107:K14	*B. nigra* Contig F	170,106	KC96001
BGH206:P22	*B. nigra* Contig F	122,354	KC96004

**Figure 2:**
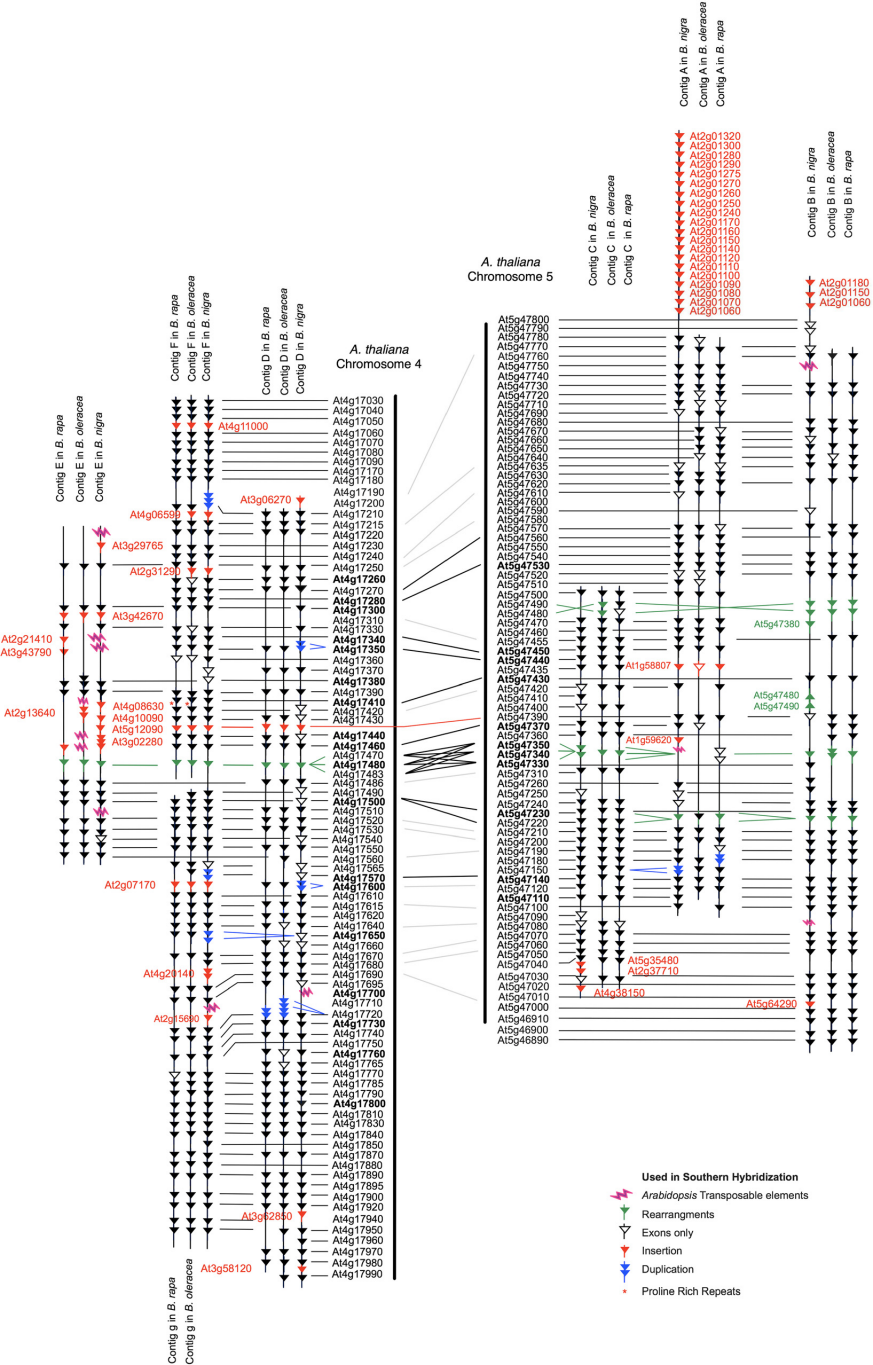
**Alignment of conserved genes between homologous regions of three *Brassica *genomes and chromosomes 4 and 5 of *A. thaliana*.**

Orthologous genes between *B. nigra* and *A. thaliana* were identified using sequence similarity, each sequenced contig was searched against the *A. thaliana* gene sequences (TAIR 10) using BLASTN and in addition were verified using *ab initio* prediction. The results are summarized in Figure 2 and show extensive conservation of gene content and order across each set of related genome segments.

**Figure 3:**
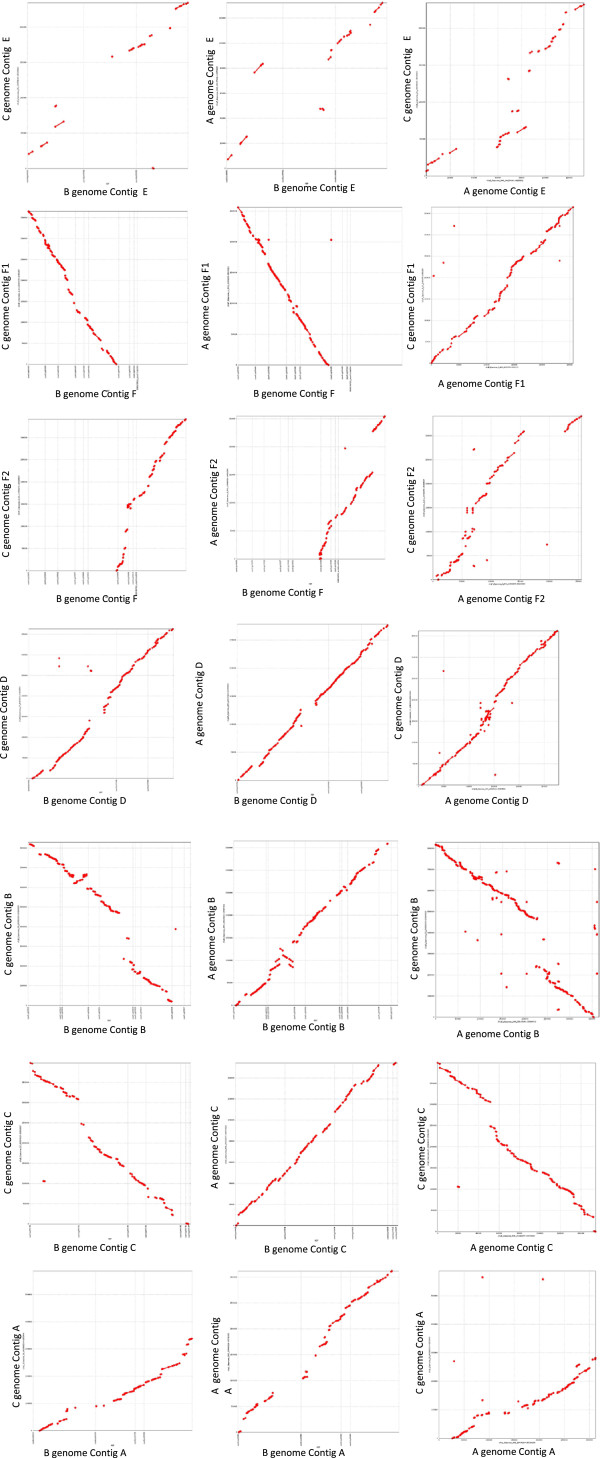
**Alignment of *B. nigra *contigs with their homologous segments in the A and C genomes as found by MUMmer.**

Five examples of gene tandem duplication were identified in *B. nigra* compared to *A. thaliana,* At5g47150 in contig A, At4g17650 and At4g17570 in contig F, and At4g17350 and At4g17600 in contig D (Figure 2). In the 6 contigs, 24 genomic insertions were detected which disrupted the collinearity (Figure 2), one of which was also a tandem repeat, At4g20140 in contig F. In five instances genes that were duplicated or triplicated in *A. thaliana* were present in only one copy in the *B. nigra* genome, apart from At5g47350 in B genome contig C where the homologous gene family was represented by two copies (Figure 2). The most significant micro-rearrangement that was specific to the B genome was observed in contig B where the region containing At5g47480 and At5g47490 was duplicated and inverted (Figures 2 and 3). Most of these rearrangements were specific to one contig, and one genome, apart from the triplicated gene family of At4g17470, At4g17480 and At4g17483, which was represented by one gene in all three genomes [32,34]. Contig E in *B. nigra* showed the highest relative incidence of gene insertion or transposition with concomitant absence of conserved gene content, with one region, between At4g17380 and At4g17480, being disrupted by the presence of six genes from unrelated regions of the *A. thaliana* genome. The same region showed a similar level of discontinuity in the C genome but the A genome although missing conserved genes showed little evidence of gene insertion. The analysis at the sequence level demonstrated that unlike the A and C genomes where an inversion, as observed in the *B. rapa* genome sequence [45] has separated contigs F and G, the B genome has maintained collinearity with *A. thaliana* chromosome 4. The set of genes from *A. thaliana* chromosome 2 at the top of contigs A and B of *B. nigra* indicate a rearrangement end point. This discontinuity in the collinearity was previously observed in *B. rapa* and indicates a break in the ancestral conserved block structure [46].

### Comparative alignment of homoeologous segments in *Brassica* diploid genomes

The overall similarity of each of the homoeologous regions of the three diploid genomes, A, B and C was compared at the nucleotide level using MUMmer as shown in Figure 3[47]. In general as expected from the observed gene content the six regions show good conservation between the three genomes. However, the sequence alignments uncover instances of genome duplications, deletions/expansions and inversions that are specific to each of the three genomes. Contigs E and F show the most distinct differences. Contig E contains a large number of genes from non-collinear regions of the genome and in addition shows further expansion through repeat element proliferation, interestingly the pattern of genome expansion is conserved between the B and C genomes; however, the types of genetic elements represented is not (Figure 2). Contig F has seen expansion in the C genome through tandem duplication of members of a gene family of proline rich genes. The C genome has more observed instances of genome expansion compared to either the A or B genomes, which is particularly notable in contig B (Figure 3 and Table 3). The remarkable expansion of this region in the C genome is due to the insertion of a large non-collinear segment (~200 kb) between At5g47070 and At5g47100.

**Table 3:** **Level of fractionation in the triplicated regions of the three *Brassica *genomes**

	**Size (bp)**	**Number of potential TEs**	**Number of retained exons (%)**	**Number of retained genic regions (%)**^**1**^		**Number of predicted genes (%)**^**2**^	**Number of *ab initio *genes**	**Gene density (gene/bp)**
**ContigA**								
Arabidopsis	221029		**388**		67			
A Genome	311929	3	190 (48.97)	46 (68.66)		29 (43.28)	72	4332.35
B Genome	258940	5	207 (53.35)	44 (65.67)		35 (52.24)	71	3647.04
C Genome	586573	32	184 (47.42)	43 (64.18)		28 (41.79)	159	3689.14
				35 genes in all 3				
**ContigB**								
Arabidopsis	341067		**496**		89			
A Genome	365330	14	270 (54.44)	55 (61.80)		41 (46.07)	82	4455.24
B Genome	258875	1	288 (58.06)	62 (69.66)		46 (51.69)	64	4044.92
C Genome	828528	42	287 (57.86)	55 (61.80)		40 (44.94)	189	4383.75
				47 genes in all 3				
**ContigC**								
Arabidopsis	182585		**243**		42			
A Genome	154252	0	174 (71.60)	34 (80.95)		25 (59.52)	32	4820.38
B Genome	175460	2	163 (67.08)	36 (85.71)		31 (73.81)	38	4617.37
C Genome	398219	14	181 (74.49)	34 (80.95)		29 (69.05)	102	3904.11
				31 genes in all 3				
**ContigD**								
Arabidopsis	327453		**456**		88			
A Genome	278470	0	243 (53.29)	64 (72.73)		42 (47.73)	66	4219.24
B Genome	386079	4	336 (73.68)	73 (82.95)		48 (54.55)	115	3357.21
C Genome	362518	4	240 (52.63)	64 (72.73)		45 (51.14)	99	3661.80
				62 genes in all 3				
**ContigE**								
Arabidopsis	112818		**222**		34			
A Genome	132246	8	92 (41.44)	17 (50.00)		14 (41.18)	38	3480.16
B Genome	159548	7	85 (38.29)	15 (44.12)		14 (41.18)	42	3798.76
C Genome	234259	10	82 (36.94)	16 (47.06)		13 (38.24)	70	3346.56
				12 genes in all 3				
**ContigF**								
Arabidopsis	387646		**503**		96			
A Genome	510517	12	366 (72.76)	80 (83.33)		65 (67.71)	121	4219.15
B Genome	450974	8	371 (73.76)	84 (87.50)		70 (72.92)	113	3990.92
C Genome	543877	37	363 (72.17)	86 (89.58)		62 (64.58)	159	3420.61
				76 genes in all 3				

1. Indicates the presence of complete and partial genes as determined by significant homology to any region of the annotated genes in *A. thaliana*. 2. Indicates homology to complete *A. thaliana* genes based on the maintenance of intron/exon structure.

### Level of gene conservation and fractionation in triplicated regions

As with previous comparative analysis conservation was inferred from the presence of significant sequence similarity between genic regions in the three genomes. However, this does not extend to the maintenance of entire gene structures and fractionated exon loss is a common feature of plant genome evolution [48]. The retention of exonic regions for the conserved *A. thaliana* genes was calculated for each *Brassica* genome contig (Table 3 and Additional file 1: Table S3). The results as shown in Table 3 indicate that for five out of the six regions the B genome has maintained a higher number of complete gene copies. For those B genome contigs homologous to *A. thaliana* chromosome 4, two of the regions have a lower level of fractionation compared to the third (74% retention of exonic regions in contigs F and D, 38% in contig E). While the A and C genomes show a similar trend, the expected stronger conservation of one of the three duplicated regions is more apparent. The two triplicated regions of the B genome studied follow the pattern of genome maintenance as observed previously for the A genome where one region, the ‘least fractionated’ (LF) is more highly conserved than either of the other two, ‘more fractionated1’ (MF1) and MF2 [45]. However, the two MF regions of the B genome appear to have incurred reduced deletion events compared to the A and C genomes as shown by a higher prevalence of gene retention (Table 3 and Additional file 1: Table S3). It has been suggested previously that higher levels of fractionation can be the result of on-going transposable element activity [49]; however, although in general the C genome shows both an expansion in genome size and repetitive element composition, this is not true for the A genome (Table 3).

### Timing of genome divergence

Synonymous base substitution rates (Ks values) were calculated for variable numbers of conserved genes across each genomic region of the three *Brassica* genomes and *A. thaliana* (Table 4). The mean values of Ks and divergence times are in agreement with values reported previously for the A and C genomes [25,33]. The divergence time of the ancestral *Brassica* genome from *A. thaliana* was estimated between ~13.9 Mya in contig A to ~17 Mya in contig D, which is similar to previous estimates of 14.5 - 20 Mya [24]. Contigs homologous to *A. thaliana* chromosome 4 in all three *Brassica* genomes were suggested to be significantly older than those homologous to chromosome 5 (p < 0.0001), which could suggest that as in many plant species the whole genome duplication event in *A. thaliana* was as a result of allopolyploidy. Previous estimates suggest *B. rapa* and *B. oleracea* diverged at around 3.7 Mya [26] and our calculations are in accordance with an average value of 3.2 Mya. The divergence time of *B. nigra* from the *B. rapa/oleracea* lineage was estimated previously based on limited available sequence data to be 7.9 Mya [25]. As detailed in Table 3 the calculated divergence times for *B. nigra* from *B. rapa/oleracea* for the target regions varied with an average of 6.2 Mya, although lower values were observed for contig E that could result from a smaller number of genes being available for comparison. It was assumed that the whole genome triplication (WGT) event observed in the B genome was shared with the A and C genomes and calculation of Ks values for conserved genes across genomic segments within each of the *Brassica* genomes corroborated this assumption, with no significant difference between the average age of divergence of the WGT (11.6 ± 3.4 Mya).

**Table 4:** Pair wise divergence time of genome segments based on synonymous base substitution rates

**Region**		**A genome**		**C Genome**		**Arabidopsis**	
	**Number of genes**	**Average Ks**	**Mya**	**Average Ks**	**Mya**	**Average Ks**	**Mya**
**B genome Contig A**	15 genes	0.18 ± 0.07	6.073 ± 2.18	0.19 ± 0.06	6.60 ± 2.04	0.42 ± 0.10	14.30 ± 3.28
**A genome Contig A**				0.09 ± 0.05	3.22 ± 1.68	0.41 ± 0.09	13.68 ± 2.91
**C genome Contig A**						0.41 ± 0.09	13.96 ± 3.16
**B genome Contig B**	25 genes	0.20 ± 0.07	6.92 ± 2.25	0.22 ± 0.06	7.36 ± 2.10	0.42 ± 0.09	14.20 ± 2.99
**A genome Contig B**				0.09 ± 0.05	3.20 ± 1.65	0.43 ± 0.10	14.44 ± 3.24
**C genome Contig B**						0.45 ± 0.10	15.04 ± 3.31
**B genome Contig C**	16 genes	0.18 ± 0.06	6.30 ± 2.01	0.18 ± 0.07	6.06 ± 2.41	0.45 ± 0.13	15.07 ± 4.45
**A genome Contig C**				0.10 ± 0.06	3.47 ± 1.85	0.43 ± 0.10	14.54 ± 3.41
**C genome Contig C**						0.43 ± 0.11	14.39 ± 3.61
**B genome Contig D**	28genes	0.20 ± 0.11	6.81 ± 3.67	0.19 ± 0.09	6.34 ± 2.96	0.50 ± 0.14	16.69 ± 4.69
**A genome Contig D**				0.10 ± 0.06	3.59 ± 1.85	0.52 ±0.18	17.40 ± 5.86
**C genome Contig D**						0.50 ± 0.18	16.99 ± 5.99
**B genome Contig E**	7 genes	0.15 ± 0.04	5.17 ± 1.46	0.14 ± 0.04	4.91 ± 1.38	0.48 ± 0.19	16.03 ± 6.44
**A genome Contig E**				0.08 ± 0.05	2.97 ± 1.51	0.46 ± 0.18	15.47 ± 6.12
**C genome Contig E**						0.46 ± 0.18	15.59 ± 6.12
**B genome Contig F**	41 genes	0.19 ± 0.05	6.46 ± 1.91	0.18 ± 0.06	6.32 ± 1.98	0.47 ± 0.14	15.93 ± 4.70
**A genome Contig F**				0.08 ± 0.03	2.76 ± 1.11	0.48 ± 0.14	16.10 ± 4.64
**C genome Contig F**						0.47 ± 0.14	15.83 ± 4.65

## Discussion

In the 1950’s when *B. juncea* replaced *B. nigra* as the mustard crop of choice in Asia, *B. nigra* was effectively abandoned with regards to crop improvement [50] and has been the subject of limited breeding, genetics and genomics research compared to the other two diploid species of U’s triangle [51]. However, the *Brassica* B genome has been recognised as a useful source of novel alleles for various traits of interest, in particular disease resistance and tolerance to abiotic stress, and numerous attempts have been made to transfer these traits to the *Brassica* A and C genomes, with varying success [9,13,14,17,52-54]. An improved understanding of the genome structure and gene composition of the *Brassica* B genome could provide insights into its relationship among the *Brassica* species and could potentially facilitate exploitation of this important resource. The current study benefited from previous research that had studied extensively the organisation of the *Brassica* A and C genome regions homologous to a 222 kb region of *A. thaliana* chromosome four, which was itself duplicated on chromosome five [44].

The *Brassica* B genome was separated previously from the A/C genome lineage in phylogenetic analyses [23,55]. Analysis of the six regions within the *Brassica* B genome compared to both the A and C genomes allowed a more accurate estimate of the timing for divergence of the two lineages, ~ 6.2 Mya (± 2.19), compared to those previously published which ranged widely from 7.9–14.6 Mya [25] to 5–10 Mya [56]. The pattern of genome rearrangements and gene deletions that differentiate the *Brassica* genomes over this period have led to the extant species. The *Brassica* B genome as for the other diploid *Brassica* genomes, retained three genomic copies of each *A. thaliana* region, reflecting the underlying whole genome triplication (WGT) event that distinguishes the Brassiceae tribe [25]. The same rate of synonymous substitution was observed between the triplicated copies of the three *Brassica* genomes and *A. thaliana* (Table 4) and similarly the intragenomic rate, (A: Ks =0.323 ± 0.12, B: Ks =0.353 ± 0.08, C: Ks =0.353 ± 0.11) across the triplicated regions within the genomes was relatively equivalent, suggesting the B genome evolved from the same paleohexaploidy event as the A and C genome. As reported previously in *B. oleracea*, *B. rapa* and *B. napus*[32,34] and shown in Figure 2 there is a high level of conservation for the composition and order of genes among the three *Brassica* diploid genomes compared to *A. thaliana*. Observed breaks in collinearity were due to predominantly minor rearrangements, such as inversions, duplications, small insertions and deletions, although one large insertion (> 200 kb) of a non-collinear segment in the C genome was found. One major chromosomal rearrangement, a large inversion relative to *A. thaliana* chromosome four, differentiated the A/C lineage from the *Brassica* B genome for one of the triplicated copies, which was also apparent from the genome sequence of *B. rapa*[45]. Genetic mapping of the B genome in *B. juncea* has certainly indicated that a number of large-scale chromosomal rearrangements may differentiate the *Brassica* A and B genomes [22], although this study also indicated that at least three of the B genome chromosomes were virtually collinear with their A genome homoeologue [22]. In addition, they identified conservation of ancestral Brassicaceae block structures across the three diploid Brassica genomes. Interestingly, as indicated in Figure 2, endpoints delineating ancestral Brassicaceae blocks, in this case between *A. thaliana* chromosomes two (block K) and five (block V), were found to be conserved between at least two of the triplicated copies in both the *Brassica* A (on linkage groups A02 – contig L, and A09 – contig B) and B genomes [45,46]. The identification of such conserved ancestral rearrangements will further assist in defining the structure of the progenitor genome of the Brassiceae tribe, which is proposed to differ from that of *A. thaliana*[57].

Although the regions studied demonstrated marked conservation relative to *A. thaliana*, each species and each genomic region were characterized by the presence of gene sequences from non-collinear regions, which were rarely shared. However, there appear to be regions that are more sensitive to such alterations and although they are common to all genomes in lacking collinearity (for example in contig E between At4g17480 and At4g17390) the changes observed were specific to each. In addition, each of the regions across the genomes varied in size (Table 3). The *B. nigra* B genome has been reported to be 632 Mb (0.65 pg), smaller than *B. oleracea* at 696 Mb (0.71 pg) but larger than *B. rapa* at 529 Mb (0.54 pg), (Johnston et al., 2004). It had previously been shown that the C genome was expanded compared to the A genome for the targeted regions, which would be in accordance with current genome estimates; however, our estimates for this region place the B genome closer in size to that of *B. rapa*.

There has been considerable interest in attempting to transfer genomic regions from the B genome into the A and C genomes of *B. napus*[4,13,14,58]; however, this has been largely unproductive due to limited or no recombination occurring between introduced B genome chromosomes and their supposed orthologues. Even the use of artificial resynthesis to develop new allopolyploids, which greatly enhances the level of recombination between the *Brassica* A and C genomes [11], has been ineffectual [59]. Yet at the genetic level [22] and as shown here at the sequence level there is marked conservation across the three *Brassica* genomes, in fact the level of karyotype and microstructure divergence across the three genomes is comparable. The question is why is aberrant homoeologous recombination so frequently observed in newly resynthesized *B. napus* (approximately 10% of the total observed recombination) but absent in equivalent resynthesized *B. juncea*[11,59]. Although it is possible that there is a genetic barrier to recombination such as that found in wheat [60] or proposed for *B. napus*[18] the inability to breakdown this control would suggest limited or no variation exists for the trait, or unlike the progenitors of wheat the B genome diploid maintains activity for a trait which has value only in the polyploid state. If the barrier is then physical, then chromosomal rearrangements not captured in previous genetic mapping studies presumably differentiate the genomes, one such example would be the inversion found here that is specific to the A/C lineage. Inversions have been proposed to have a significant impact on plant evolution [61] and could have lead to the reproductive isolation and speciation of the B genome.

**Figure 4:**
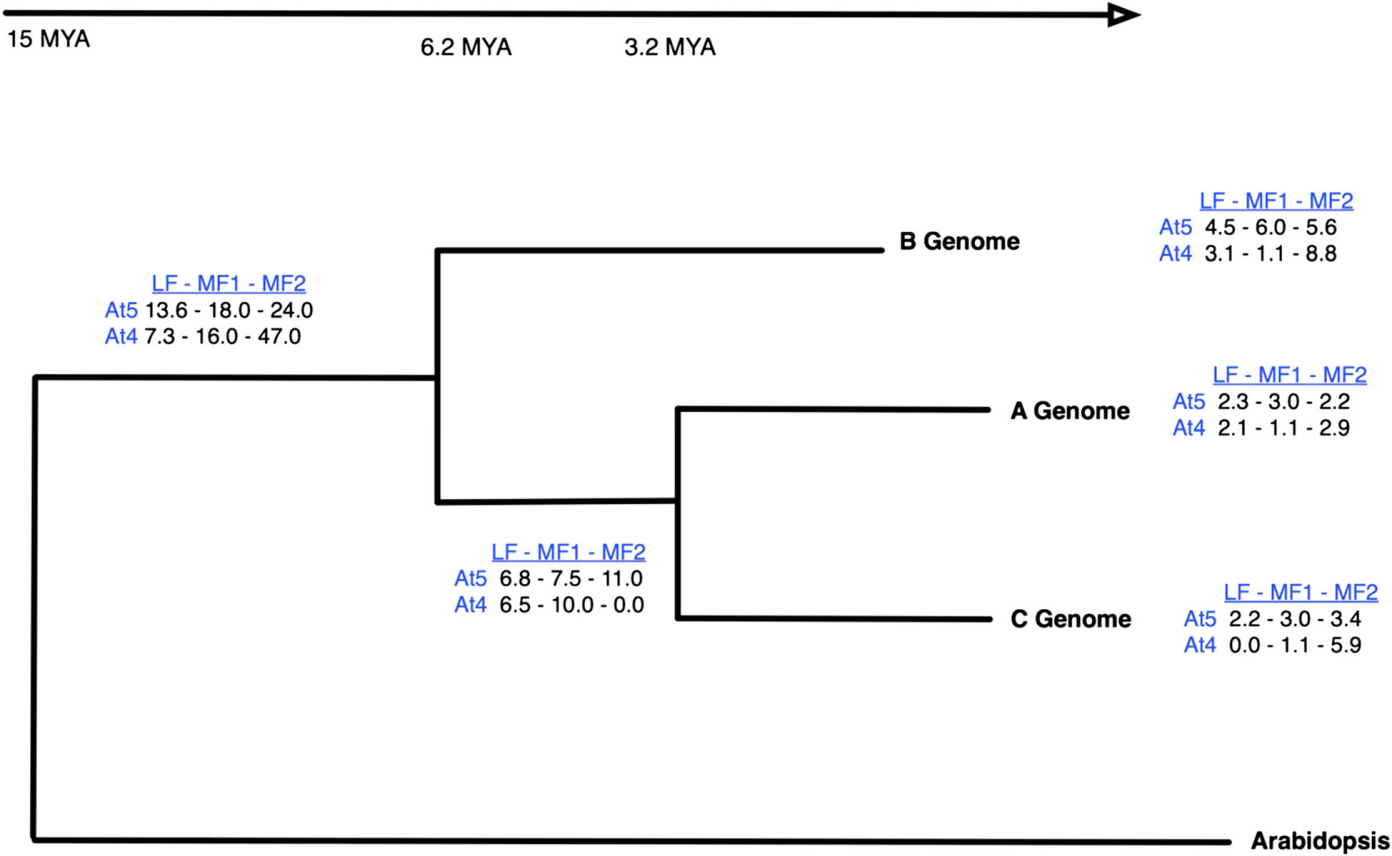
**Phylogenetic tree of the *Brassica *B and A/C lineage.** The percentage of fractionation for the three sub-genomes at each node is specified. The contigs are ordered according to the overall level of fractionation from least (LF) to most fractionated (MF2) (Table 3).

Although the current analyses focuses on two triplicated regions of the *Brassica* B genome, it provides insights into the gene retention pattern across the wider genome, since the A genome regions studied here reflect the pattern found in the *B. rapa* genome sequence [45]. The publication of the first *Brassica* genome sequence, that of *B. rapa*, provided extensive evidence of gene fractionation following the WGT and perhaps most interestingly due to bias in gene retention in the three genomic copies led to the two-step polyploidy hypothesis with fractionation between steps [45]. This hypothesis, in which the paleohexaploid was formed from the hybridisation between a diploid and an established tetraploid, has been rigorously tested since, with the retention of exonic regions being studied in detail for the A genome [62] and indicating that deletions are the major mode of gene inactivation and hence evolutionary drive in *Brassica* species. For the studied regions, the B genome appears to follow the same gene fractionation pattern as the A genome with one of the three genomic copies showing a higher level of conservation (Figure 4; Additional file 1: Table S3). The observed level of gene loss or fractionation for the triplicated regions at each important branch point in the evolution of the studied genomes is indicated in Figure 4. The highest percentage of fractionation appears to have occurred prior to the separation of the three genomes that is where gene loss is shared across all three genomes. Although the general pattern of fractionation holds true species-specific differences can be created through the types of genes that are maintained. For example in the regions homologous to *A. thaliana* chromosome five, no copies of two annotated disease resistance genes, At5g47250 and At5g47260, are found in the A and C genomes, yet multiple full or partial copies are maintained for each in the three homologous B genome regions. In addition, local insertion of a further annotated disease resistance gene (At1g59620) in this region of the B genome for contig A indicates a resistance cluster specific to the B genome.

## Conclusions

Analysis of homologous regions of three closely related Brassica diploid genomes has identified extensive conservation of gene content and order, which is not reflected in the established phylogenetic relationship of the three species. The genome of *B. nigra*, based on its inability to establish effective pairing structures with the A and C genomes, might have been expected to have undergone more rearrangements at the micro-level, suggesting perhaps that major chromosomal events such as the observed inversion could have played a significant role in the speciation of the B genome. The B genome is likely to be an excellent source of novel genes for a number of important traits and capturing this diversity through genome sequencing could prove important for future crop improvement.

## Methods

### Construction of BAC library

Seedlings of a doubled haploid *B. nigra* (DH No100, provided by Alison Ferrie, National Research Council of Canada Plant Biotechnology Institute (NRC-PBI), Saskatoon) were grown in a controlled environment cabinet at a constant 20°C with 16 hour photoperiod until 6–8 leaf stage. Plants were placed in the dark for 3 days prior to tissue harvest, leaf tissue was flash frozen in liquid nitrogen and shipped to BioS&T, Montreal, Canada (http://www.biost.com/) for library construction. Large insert genomic DNA was ligated into the *Hind*III site of the pIndigoBAC-5 vector (Epicentre, Madison, WI, US). The library clones are prefixed “BGH” and are arrayed in 222 384 well plates, with an average insert size of 130 kb, representing almost a 20 X coverage of the *Brassica* B genome which has an estimated size of 632 Mb based on flow cytometry [40].

### Primary BAC library screening

The library was gridded in replicate on five 22.2 cm^2^ Hybond™-XL (GE Healthcare Uppsala, Sweden) membranes and screened by colony hybridisation using probes of seventeen genes from the *A. thaliana* 222 kb target region of chromosome 4 [29] to identify homologous clones (Table 1). Preparation of the *A. thaliana* gene specific probes and their sequence are detailed in O’Neill and Bancroft (2000). These probes were labeled by the random priming method using the RediPrime II kit (GE Healthcare Uppsala, Sweden) according to the manufacturer’s instructions. Hybridisation was carried out at 65°C for 16 h in QuikHyb Hybridisation Buffer (Agilent Technologies Santa Clara, CA) according to the manufacturer’s instructions. The membranes were washed twice for 10 min at 65°C followed by three washes for 10 min at room temperature in 2 × SSC and 0.1% SDS.

### BAC DNA preparation

DNA from BAC clones identified in the primary screen was prepared in one of two ways: for DNA digestion, HICF and BAC end sequencing, BAC DNA was prepared using standard alkaline lysis [63]. For full length sequencing, DNA was isolated using the Qiagen Large Construct Kit (Qiagen, Valencia, CA) according to the manufacturer’s instructions. The integrity and size of the clones was confirmed by digestion with *Not*I and resolution by Pulse Field Gel Electrophoresis (PFGE) on a 1% agarose gel in 0.5 × TBE at 120° angle for 16 hours at 14°C with a 0.1 – 40.0 second switch. The sizes of the fragments were estimated using the Lambda Ladder PFG and MidRange II PFG markers (NEB Ipswich, MA).

### Southern blot analysis

Purified BAC DNA was digested with *Hind*III enzyme and separated on 1% agarose in 1 × TAE at 5V for 18 hours. The digested DNA was blotted onto Hybond XL membranes and probed as above. Hybridisation was done at 65°C for 16 h in modified Church buffer [64] and the membranes were washed twice for 10 min at room temperature in 2 × SSC and 0.1% SDS.

### BAC sequence analyses

BAC end sequencing was completed for 1,110 positive clones using modified BigDye Terminator v.3.1 and BigDye Xterminator purification kit protocols (Life Technologies Carlsbad, CA). Sequencing reactions were run on an AB 3730×l at the NRC DNA Technologies Laboratory in Saskatoon. The resultant sequence data was compared to itself and to *A. thaliana* using BLASTN with default parameters and an E value cut-off of 1 × E-10. Full length BAC sequences were generated with Roche 454 Flex sequencing also at NRC. The 15 BACs were each indexed and sequenced in one half of a 454 plate. The sequences were assembled into large contigs using Newbler v 2.6 (Roche Diagnostics).

### SNaPshot fingerprinting

The fingerprinting reaction was performed following Luo et al., (2003) with minor modifications recommended by the Arizona Genomics Institute. The BAC DNA was incubated with the digestion and labeling mix for one hour at 37°C and labelling was performed for one hour at 65°C. The size standard geneScan LIZ-1200 (size range from 20 to 1200 bp) was added to each sample prior to loading on an AB 3100 for capillary electrophoresis using the DS-02 dye set. Peak height, area and sizes were collected by GeneMapper® software v3.7 and converted into FPC readable format after editing by the GenoProfiler® v2.1 (http://wheat.pw.usda.gov/PhysicalMapping/; [41]).

### Data editing operations and FPC contig assembly

The editing process included removal of vector bands and removal of clones for which the reaction failed, lacked an insert or were suggestive of cross contamination [65]. The files generated by GenoProfiler were transferred to FPC v9.3 (http://www.agcol.arizona.edu/software/fpc; [42]). Initially, a tolerance value of 3.0 and a Sulston cut off score of 1 × 10^-35^ was used to assemble contigs automatically, the stringency was later reduced as described in the Results section. “DQer” function of FPC was used to reassemble contigs with more than 10 Q clones. The resulting contigs were merged by the “End to End” auto merge function with a minimum of two matching ends. The remaining singletons were merged to form contigs by the “Auto merge/add” function. The fingerprints of clones in the merged contigs were then analysed manually and compared to contigs assembled based on Southern blot analyses to finalize the contigs [31].

### Sequence analyses

The genome assembly of *B. rapa* (http://brassicadb.org) and preliminary assembly of *B. oleracea* (TO1000 sequencing consortium, unpublished data) were used to identify extended A and C genomic regions equivalent to the sequenced B genome regions. The *A. thaliana* genome sequence (TAIR-10; http://www.arabidopsis.org) was compared to the *Brassica* sequences using BLASTN with default parameters and an Expect value of 1 × E^-10^. GlimmerHMM [66] was used to predict genes using training data from *A. thaliana*. MUMer and NUCmer were used to align contigs of the AC genome with those of the B genome and the alignments were visualised using MUMmer plot [47].

### Calculation of Ks values and phylogenetic tree

Homologous predicted cDNA sequences from the *Brassica* genomes and *A. thaliana* were aligned using ClustalW version 1.83 [67]. Ks and Ka values were calculated in a pair wise model using the CODEML program in the PAML package version 4.4 [68]. The commonly adopted estimate of mutation rate of 1.5 × 10^-8^ synonymous substitutions per site per year [69] was used to estimate the divergence time of the three genomes.

The alignments of the four orthologous gene sets were trimmed for regions with gaps and missing data using trimAl [70]. The alignment of 126 gene sets were concatenated using the Phyutility software [71], building a total alignment length of 120,936 bps which was then used for the phylogenetic analysis performed by the FastTree2.1.3 [72] and processed using Dendroscope [73]. Robustness of phylogenetic inference was assessed by the bootstrap analysis approach for 1000 reassembled alignments.

## Competing interests

The authors declare that they have no competing interests.

## Authors’ contribution

ZKN performed the research and analysed the data. ZKN and IAP designed the research and wrote the manuscript. TH prepared, quality assessed and carried out initial screening of the BAC library. IB and CMO provided the Arabidopsis probes and edited the manuscript. AGS assisted with sequencing of the BAC clones. All authors read and approved the final manuscript.

## Supplementary Material

Additional file 1: Table S1 Overlapping BACs based on Southern Hybridization analysis. +: positive hybridization with the gene specific probe, -: no hybridization. Highlighted cells are the fully sequenced BACs. **Table S2.** Overlapping BACs based on the FPC analysis. **Table S3.** Comparison of the level of exon retention across the three *Brassica* genomes. Click here for file
